# Effectiveness of various methods to reduce aflatoxin M1 levels in milk, a systematic review

**DOI:** 10.1016/j.fochx.2024.101737

**Published:** 2024-08-13

**Authors:** Yeganeh Mazaheri, Parisa Shavali-gilani, Nabi Shariatifar, Alireza Bakhtiyari, Zahra Hadian, Nader Akbari, Narges Abdoli, Parisa Sadighara

**Affiliations:** a Department of Environmental Health Engineering, Division of Food Safety and Hygiene, School of Public health, Tehran University of Medical Sciences, Tehran, Iran; b Department of Food Technology Research, National Nutrition and Food Technology Research Institute, Faculty of Nutrition Sciences and Food Technology, Shahid Beheshti University of Medical Sciences, Tehran, Iran; c Iranian Food and drug administration, Tehran, Iran; d Ministry of Health and Medical Education, Tehran, Iran

**Keywords:** Aflatoxin M1, Milk, Aflatoxin B1, Feed, Probiotics

## Abstract

The numerous strategies have been conducted worldwide to mitigate the presence of these hazardous toxins. In this systematic study, these researches are summarized. The search of this study was done with keywords aflatoxin M1, AFM1, reduce, decrease, mitigation, prevent, prevention, and milk in databases without a time limit. A total of 49 manuscripts were carefully reviewed, and their data were extracted. Some interventions focused on modifying animal rations, aiming to reduce AFM1 in milk. Some were applied directly to the animals. In this method, which was done more than other research interventions, some toxin binders are used as feed additives. The third type of intervention consisted of measures that were taken directly on the milk itself. Among the three types of interventions, the use of toxin binders in animal feed was more practical and effective.

## Highlights

• One of the prominent milk mycotoxins is AFM1
• The most common method for reducing AFM1in milks is using absorbent in feed
• Teaching farmers to toxin binders is one effective methods to reduce AFM1

## 1 Introduction

Among mycotoxins, aflatoxin contaminates more food and agricultural products than others (Moradian, Faraji, & Davood, 2024). Aflatoxins, produced by Aspergillus fungi, constitute a group of mycotoxins known to induce various health complications, including carcinogenesis, malformations, and immunosuppression. They are also contributing to significant economic losses (Acaroz, 2019; Admasu et al., 2021). These mycotoxins lead to a decrease in appetite, weight and milk production of animals (Queiroz, Han, Staples, & Adesogan, 2012). Also, aflatoxins increase the sensitivity of livestock to infectious diseases (Xiong, Wang, Nennich, Li, & Liu, 2015). The amount of these toxic compounds in food should be reduced as much as possible (Sadighara, Basaran, Afshar, & Nazmara, 2024). Therefore, it becomes crucial to implement strategies aimed at mitigating the adverse health effects and economic repercussions associated with aflatoxins (Pate, Paulus Compart, & Cardoso, 2018). There are four types of aflatoxin B1, B2, G1, and G2, and the most contamination of food used by humans and animals is with aflatoxin B1(AFB1) (Bhardwaj et al., 2023).

In a study conducted in Tunisia, it was found that 84.4% of feed samples were contaminated with AFB1 (Abbès, Salah-Abbès, Bouraoui, Oueslati, & Oueslati, 2012). Similarly, in Kenya, during the 2006–2007 sampling period, 86% of animal feed samples were found to be contaminated with AFB1 (Kang'ethe & Lang'a, 2009). Although this mycotoxin is typically present in trace amounts, its significance lies in its exceptionally high toxicity. Because AFB1 is a small molecule, it is quickly absorbed in the digestive system(Rodrigues et al., 2019). The liver is target of AFB1, leading to severe hepatoxicity and liver cancer (Sadighara & Ghanati, 2022; Ye et al., 2024). In the liver, AFB1 undergoes a transformation into an intermediate reactive epoxide metabolite. Then it is hydroxylated and form aflatoxin M1 (AFM1). After hydroxylation in lactating animals, it is secreted into milk (Stella et al., 2024). AFB1 appears very quickly and after 15 min in the form of its metabolite, aflatoxin M1 (AFM1), in the blood. Six hours after feeding a diet contaminated with AFB1, AFM1 appears in milk(Rodrigues et al., 2019). In a study conducted on goats revealed the presence of AFM1 in milk within one hour after oral administration of AFB1-contaminated food (Battacone, Nudda, Rassu, Decandia, & Pulina, 2012). One of the most important contaminants of milk is aflatoxin M1(AFM1), which is resistant to heat (Admasu et al., 2021). This mycotoxin is not reduced during sterilization and pasteurization in milk (Liu et al., 2023). According to the International Agency for Research on Cancer, both mycotoxins are carcinogenic (Firmin, Morgavi, Yiannikouris, & Boudra, 2011).

In addition to milk, AFM1 is detected in various dairy products such as yogurt, baby food, cream, and cheese (Xiong, Wang, Zhou, & Liu, 2018a). The American Food and Drug Administration declares the permissible limit of AFM1in milk at 0.5 μg/kg and the permissible limit of aflatoxin B1(AFB1) in animal feed at 20 μg/kg (Jiang et al., 2018b). Babies and children are very sensitive to AFM1, and its permissible limit in infant food is 0.025 μg/kg (Admasu et al., 2021).

The prevalence of AFM1 in milk can be attributed, in part, to a lack of awareness among livestock farmers regarding the risks associated with the consumption of moldy feed (Anyango et al., 2021). Unfortunately, most farmers do not know much about this toxic compound in milk. In a questionnaire survey, only 32% of farmers believed that it is a toxic compound (Admasu et al., 2021).One of the most serious food safety problems is the presence of AFM1 in milk (Abbès et al., 2013; Wakade, Ingole, Bharucha, & Nagvekar, 2019). Knowing an effective and practical method to reduce this dangerous AFM1 in milk will prevent economic losses and guarantee the safety of milk. There are different methods to reduce AFM1 in milk. These strategies are classified into three biological, physical and chemical methods (Nikmaram, Brückner, Cramer, Humpf, & Keener, 2023). These methods may target animal feed, live animals, or the milk itself (Anyango et al., 2021). In this systematic study, the strategies used to reduce this mycotoxin in milk are presented and their efficiency and safety are discussed.

## 2 Method

### 2.1 Search strategy

The keywords were chosen based on a preliminary review of the texts. The search formulation was designed in this way (aflatoxin M1 OR AFM1) AND (reduce or decrease or mitigation or prevent or prevention) AND milk. The search was conducted on September 3, 2023, in two important databases, PubMed and Scopus.

### 2.2 Inclusion and exclusion criteria

The inclusion criteria for this research included manuscripts that had at least one strategy designed to reduce the amount of AFM1 in milk. Based on the evidence in the literature, the strategies included three types. The most common strategy was to use toxin binders. The next case was the actions taken on the milk itself and the third case was the actions taken on the animal feed. Manuscripts that only evaluated the amount of AFM1 in milk or the amount of AFB1 in livestock rations were excluded from this systematic study. Furthermore, review papers and manuscripts on human milk were excluded from this systematic review.

## 3 Results

### 3.1 The result of search process

**Fig. 1:**
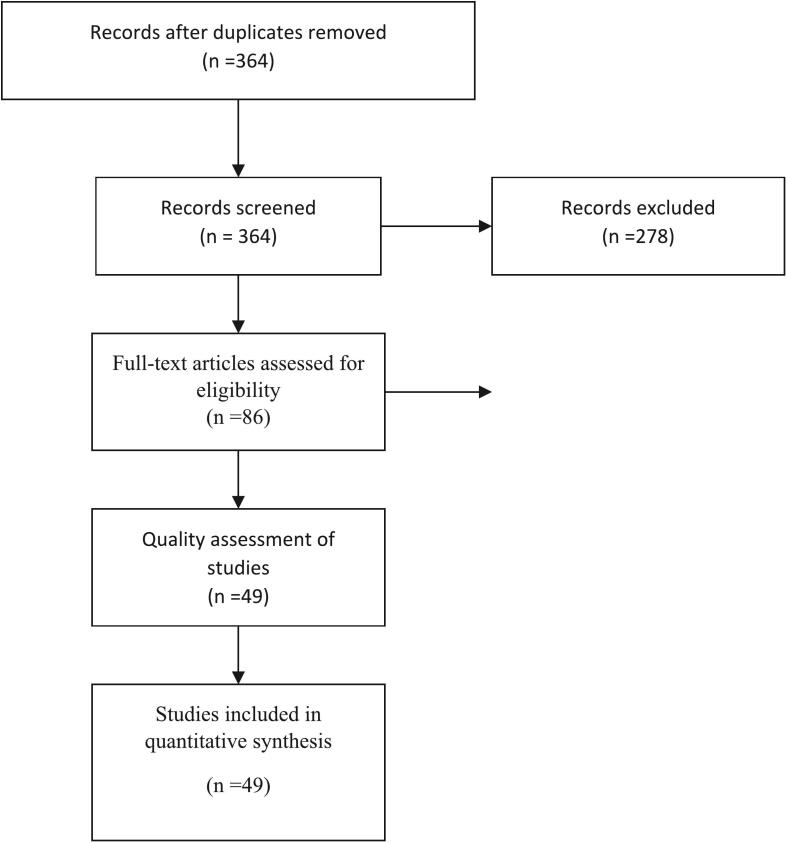
The diagram of study.

According to the determined keywords, the search was done by two members of the team. Manuscripts were entered into endnote software and duplicates removed. First, a preliminary assessment was done. The title and abstract of the manuscripts were studied. This evaluation was done according to the inclusion and exclusion criteria. The study path can be seen in Fig. 1. 364 manuscripts were initially evaluated. 86 manuscripts were fully evaluated. 49 manuscripts were selected for data extraction.

### 3.2 Data extraction

Manuscripts that investigated the reduction of AFM1in milk belonged to 22 countries. Some countries had several investigations. This geographical distribution shows the importance of the issue and also indicates that this health problem exists in many geographical areas (Table 1, Table 2, Table 3). In this study, interventions were categorized into three groups. Two groups of interventions were performed on live animals and their diets. Another intervention was done on drinking milk.

**Table 1:** Data extraction from studies done on feed.

**Type of intervention**	**Results**	**Country**	**Author/ date**
Ammonization of feed	Reducing the amount of milk AFM1Toxins	USA	Bailey/1994
Ammonization of feed (cottonseed)	Reducing the amount of milk AFM1Toxins	USA	Price/1982
Ammonization of feed (peanut cakes)	Reducing the amount of milk AFM1Toxins	France	Fremy/1987

**Table 2:** Data extraction from studies done on animals.

**Type of intervention**	**Results**	**Country**	**Author/ date**
Feeding for 30 day with AFB1 Feeding for 30 day with AFB1 + clay Feeding for 30 day with AFB1 + clay + *Saccharomyces cerevisiae* fermentation products	The amount of AFM1 in three groups 1, 2 and 3 was 0.75, 0.45, 0.40 μg/kg in milk, respectively	Brazil	Jiang/2018
Feeding for 42 day with basal diet Feeding for 42 day with Bentonite Feeding for 42 day with Bentonite + *Saccharomyces cerevisiae* cell wall Feeding for 42 day with *Saccharomyces cerevisiae* cell wall	The lowest amount of AFT was observed in the *Saccharomyces cerevisiae* cell wall group	Iran	Mirzaei/2020
Feeding with AFB1 (150 μg/ kg)+ Sodium bentonite	Reducing AFM1 by 65%	USA	Diaz/2004
Training of ranchers to use Toxin Binder	The amount of AFM1 was less than the permissible limit	Sweden	Anyango/2021
Feeding for 30 day with AFB1+ algae+ bentonite	No definite confirmation has been reached in this regard	EU	Bampidis/2019
Feeding for 30 day with AFB1+ saponite-rich bentonite	The amount of AFM1 is below the limit	Italy	Carraro/2013
Feeding for 21 day with AFB1+ modified *Saccharomyces cerevisiae* cell wall	Faecal excretion of AFM1 and AFB1 increases	France	Firmin/2011
The use of absorbent (Solis Mos)in animal feed for 9 week	Leading to an 35.2% reduction in the amount of AFM1	China	Xiong/2018
Feeding for 45 day with AFB1+ bentonite+ exal	Reduction effects in the amount of AFM1 were observed from the eighth day of treatment	India	Wakade/2019
Feeding for 30 day with AFB1+ CuraTox-FS	Reducing AFM1 by 55–72%	India	Korgaonkar/2017
Feeding for 20 day with AFB1+ Calcium propionate	Calcium propionate leads to reduced toxin transfer	Pakistan	Ali Tipu/2021
Vaccination of cattle against AFB1	50% of vaccinated animals had high antibody titers	Italy	Giovati/2014
Vaccination of cattle against AFB1	The amount of AFM1 in the milk of vaccinated animals was below the permissible limit	Italy	Polonelli /2011
Vaccination of cattle against AFB1	70% reduction of AFM1 in milk in vaccinated cows	Egypt	Roshdy/2020
Feeding for 28 day with AFB1+ 3 adsorbents(MTB, NovasilPlus, Solis)	The effectiveness of NovasilPlus, Solis and the lack of effectiveness of MTB-100	USA	Kutz/2009
Feeding with AFB1+ yeast(*Pichia kudriavzevii, Kluyveromyces marxianus, and K. marxianus*)	Reducing AFM1 by 72.08%	Thailand	Intanoo/2020
Feeding for 28 day with AFB1+ 3 adsorbents(clinoptilolite)	It is both economical and effective	Greece	Katsoulos/2016
Feeding for 14 day with AFB1+ bentonite	Reducing AFM1 by 30–43%	Kenya	Kemboi/2023
Feeding for 14 day with AFB1+ sodium bentonite Feeding for 14 day with AFB1+ yeast oligosaccharides	A positive effect of sodium bentonite was observed	USA	Kissell/ 2012
Feeding for 45 day with AFB1+ adsorbents(NovasilTM Clay)	34% reduction of AFM1 at 0.6% binder concentration	Kenya	Kuboka/2022
Feeding for 45 day with AFB1+ adsorbents(calcium montmorillonite Clay)	A positive effect of calcium montmorillonite bentonite was observed	USA	Maki/2016
Feeding for 28 day with AFB1+ 3 toxin binder (Mycosorb, Fixar Viva, and T5X)	Mycosorb = 47% Fixar Viva = 39% T5X = 56%	Pakistan	**Naveed/2018**
Feeding with AFB1(75 μg/kg) low-clay diet Feeding with AFB1(75 μg/kg) high-clay diet	The effect of high-clay group was more than low-clay	South Korea	Queiroz/2012
Feeding with AFB1(150 μg/ kg) + Toxfin(Sepiolite + bentonite +companion clays) Feeding with AFB1+ Elitox(enzymes +HSCAS + biopolymers + vitamin C + natural extracts)	Toxfin: 49.57 Elitox: 19.49	Pakistan	Ullah/2016
Feeding with AFB1(200 μg/ kg of feed)+ sodium calcium aluminosilicate	leading to an 86.9% reduction in the amount of AFM1	USA	Smith/1994
Feeding with AFB1(100 μg/ kg of feed) + adsorbents(3 clay concentration)	Positive effects were observed in three concentrations.	Ukraine	Sulzberger/2017
Feeding with AFB1(40 μg/ kg of feed) + aluminosilicate Feeding with AFB1(40 μg/ kg of feed) + yeast cell wall glucomannan	No positive effects were observed regarding yeast cell wall glucomannan	México	Rojo/2014
Feeding with AFB1(40 μg/ kg of feed)+ Activated Carbons	A positive effect of Activated Carbons was observed	Italy	Galvano/1996

**Table 3:** Data extraction from studies done on milk.

**Type of intervention**	**Results**	**Country**	**Author/ date**
Incubation of milk with Lactobacillus acidophilus	80% AFM1 reduction after 48 h	Iraq	Ahmad2022
Incubation of skimmed milk with *Bifidobacterium lactis*	37% AFM1 reduction at 4 °C	Brazil	Bovo/2012
Incubation of skimmed milk with *S. cerevisiae*(a) Incubation of skimmed milk with LAB pool(b) Incubation of skimmed milk with LAB pool+ *S. cerevisiae*(c)	(a):92% AFM1 binding after 60 min (b): 11% AFM1 binding after 60 min (c): 100% AFM1 binding after 60 min	Brazil	Corassin/2013
Incubation milk with 0.08 ppb AFM1 + *S. cerevisiae*(a) Incubation milk with 0.23 ppb AFM1 + *S. cerevisiae*(b)	(a):100% reduction after 40 min (b): 81% reduction after 80 min	Iran	Foroughi/2018
Incubation of milk with Lactic acid bacteria	Reduction of toxins in milk up to 95%	Croatia	Kuharić/2018
Incubation of milk with Peroxidase	Reduction of toxins in milk up to 60%	China	Liu/2023
Thermoultrasound treatment of milk for 10 min	Effective reduction of AFM1 was observed	Mexico	Hernández-Falcón/2018
Incubation milk with AFM1 + *S. cerevisiae* Incubation milk with AFM1 + three types of lactic acid bacteria Incubation milk with AFM1 + three types of lactic acid bacteria+ *S. cerevisiae*	The greatest effect was observed in *Lactobacillus helveticus*	Brazil	Ismail/2016
Irradiating UV rays to contaminated milk	It leads to the reduction of AFM1 up to 89%.	USA	Yousef/1986
Incubation of milk with Lactic acid bacteria	Effective results were not obtained	Turkey	Kabak/2008
Incubation of milk with AFM1 + *Lactobacillus rhamnosus* Incubation of milk with AFM1 + *Lactobacillus plantarum* Incubation of milk with AFM1 + Saccharomyces boulardii	Effective reduction of AFM1 was observed in all three types of treatment	Iran	Khadivi//2020
Irradiating UVC rays to contaminated milk	It leads to the reduction of AFM1 up to 50%.	New Zealand	Nguyen/2022
Incubation of milk with AFM1 + *Lactobacillus rhamnosus* Incubation of milk with AFM1 + *Pediococcus acidilactici* Incubation of milk with AFM1 + *P. pentosaceus* Incubation of milk with AFM1 + *Saccharomyces cerevisiae* Incubation of milk with AFM1 + *S. boulardii* Incubation of milk with AFM1 + *Kluveromyces marxianus*	Positive effects of incubation with *P. pentosaceus* and K. marxianus were observed.	Argentina	Martínez/2019
Incubation of raw milk with AFM1 + Kaolin Incubation of raw milk with AFM1 + Ca- bentonite	Positive and dose-dependent effects were observed in both treated groups.	Egypt	Moussa/2019
Incubation of milk with AFM1 + *P. pentosaceus* Incubation of milk with AFM1 + *Saccharomyces cerevisiae*	Both of these bacteria had the power to destroy AFM1	Iran	Namvar Rad/2018
Cold plasma	Decomposition of AFM1up to 78%	New Zealand	Nguyen/2022
Cold plasma	The amount of AFM1 decreased with the increase of exposure up to 5 min	Germany	Nikmaram/2023
Incubation of milk with AFM1 + β-cyclodextrin	Decomposition of AFM1up to 39.1%	Slovakia	Simko/2022

Table 1 included studies that were directly applied to animal feed. The other strategies to reduce the exposure to AFM1 is to use a series of food additives in the ration of animals to reduce absorption of AFB1 in the digestive tract of animals (Firmin et al., 2011). Table 2 shows the food additives used in livestock diets and the strategies used to increase the immunity of livestock. Table 3 details the strategies employed to diminish AFM1 levels in milk post-milking from animals.

## 4 Discussion

### 4.1 Interventions made on feed

It is estimated that 25% of agricultural products are contaminated with aflatoxins (Pate et al., 2018). Three studies had strategies to reduce AFB1 directly on animal feed, and ammonia was used in all three studies. In the Price, Lough, and Brown (1982) study, cottonseed was treated with ammonia. In this study, dairy cows were fed cottonseed treated with ammonia, resulting in a notable decrease in AFM1 levels in the milk over the 25–27 day duration. Conversely, when the feed without added ammonia was reintroduced, the AFM1 levels in the milk increased. A similar study was conducted by Fremy et al. (1987). One of the animal feed is peanut cake, which is a by-product of peanuts after oil extraction (Dias et al., 2018). The peanut cake was treated with ammonia. It was observed that the amount of milk AFM1 was lower in cows that received treated food (Fremy, Gautier, Herry, Terrier, & Calett, 1988). Of course, the US Food and Drug Administration did not approve this method (Price et al., 1982).

### 4.2 Interventions made on livestock

**Fig. 2:**
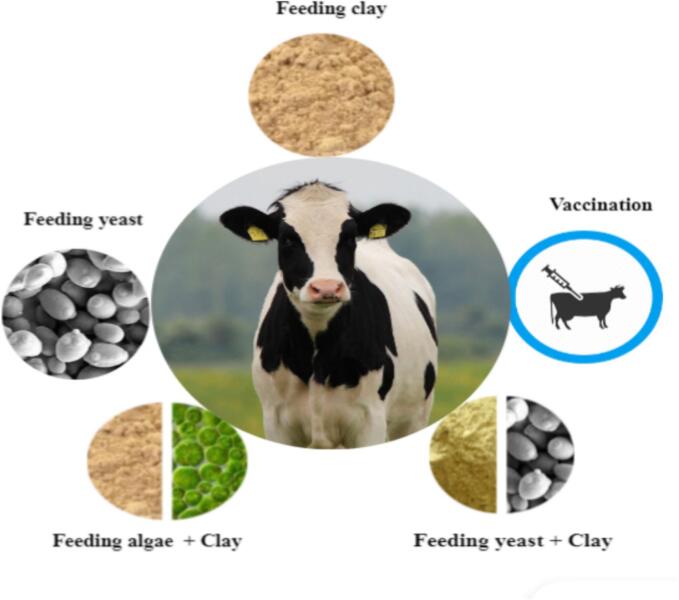
Interventions made on livestock for reducing AFM1 in milk.

Fig. 2 illustrates a summary of interventions performed on livestock to reduce AFM1 in milk. Certain feed additives, such as toxin binders, possess the capability to bind to AFB1, and prevent its absorption from the digestive system (Kissell, Davidson, Hopkins, Smith, & Whitlow, 2013). The toxin binders in animal food bind to AFB1 and prevent their absorption from the digestive system (Wakade et al., 2019). In an intervention study, livestock farmers were trained and encouraged to utilize toxin binders. As a result, there was a decrease in the levels of AFM1 and an increase in milk production (Anyango et al., 2021).

For this purpose, according to the extracted data, the primary material added to the livestock diet was clay. The clay used in animal feed usually include bentonite, aluminosilicates, zeolite, etc. (Hussein, Abdo, Hassan, Farghali, & Farghaly, 2023). Besides inhibiting the absorption of AFB1, these compounds also serve to impede the absorption of other hazardous substances like heavy metals, nitrates, nitrites, and dyes (Montayeva, Montayev, & Montayeva, 2023).

In the Carraro et al. (2013) study conducted in Italy, various bentonites were fed to livestock for investigation. The best efficiency was observed in saponite-rich bentonite (Carraro et al., 2014). Positive effects of reducing AFM1 due to the use of bentonite alone have also been seen (Kemboi et al., 2023; Kissell et al., 2013). Similar outcomes were recorded in the Ullah et al. (2016) study. Goats were fed with two types of commercial sorbents. The absorbent that had bentonite in its composition had positive effects (Ullah et al., 2016). Diaz et al. (2004) found that sodium bentonite at a concentration of 1.2% of the diet led to a 65% reduction of AFM1 in milk (Diaz et al., 2004). Furthermore, the study by Wakade et al. (2019) utilized a daily dose of 50 mg of an adsorbent containing exal and bentonite, demonstrating a reduction in AFM1 transfer to milk without causing any toxic effects on livestock (Wakade et al., 2019). In some cases, other compounds such as algae were also used along with toxin binder. The European Union panel has not yet approved the efficacy of algae interspaced bentonite as feed additives in animal feed (Additives et al., 2019).

In the Katsoulos, Karatzia, Boscos, Wolf, and Karatzias (2016) study, 200 g of clinoptilolite along with AFB1 were administered to cows for 7 days. Compared to the control group, the positive effects of this absorbent were confirmed (Katsoulos et al., 2016). Clinoptilolite is a type of natural zeolite (Hernández-Martínez et al., 2023).

In the Makki et al. (2016) study, dairy cows were fed with calcium montmorillonite in two doses, 6.0 and 12.1 g/kg. The amount of AFM1 decreased by 55% and 68% respectively in milk (Maki et al., 2016). Montmorillonite is one of the clays with three layers, which has the ability to absorb compounds in all three layers. In addition to AFB1, it demonstrated effectiveness in absorbing heavy metals (Sulzberger, Melnichenko, & Cardoso, 2017). Similar to this result, in the Sulzberger et al. (2017) study, positive effects of clay were noted at a concentration of 2 % of the weight of dry matter (Sulzberger et al., 2017). In this study, three compositions of vermiculite, nontronite, and montmorillonite were identified in this clay by x-ray (Sulzberger et al., 2017). In the study of Queiroz et al. (2012), the effects of reducing aflatoxin in the milk of cows fed with clay were observed 10 to 12 days after feeding (Queiroz et al., 2012). The clay used was a commercial type with montmorillonite hydrated sodium‑calcium-aluminosilicate formulation (Queiroz et al., 2012). The adsorbent (Mastersorb) was employed in two concentrations of 0.6% and 1.2% in the livestock diet in a study. 34–45% reduction of AFM1 in milk was observed (Kuboka, Njue, Mutua, Grace, & Lindahl, 2022). The exact composition of Mastersorb was not determined in this study. But, the authors declared that it is based on clay. In the study of Smith et al. (1994), it was observed that the use of sodium calcium aluminosilicate in the diet of goats receiving AFB1 leads to the reduction of AFM1 in milk effectively and significantly (Smith et al., 1994).

However, it is worth noting that clays in high concentrations may lead to a reduction in the absorption of nutrients and minerals from the digestive system (Jiang et al., 2018a). Therefore, the appropriate concentration of clays should be calculated to avoid potential negative impacts on nutrient and mineral absorption.

Some absorbents contain yeast in addition to clay, which usually enhances the efficiency of mycotoxin removal. In the study of Xiong et al. (2018), cows were fed Solis Mos absorbent for 9 weeks. Solis Mos is an absorbent containing sodium montmorillonite, vitamin E, and yeast. It was observed that, besides reducing toxin transfer, antioxidant factors such as glutathione peroxidase, superoxide dismutase, and IgG levels increased (Xiong, Wang, Zhou, & Liu, 2018b). Therefore, this adsorbent also exhibits antioxidant properties, which is due to yeast in accordance with previous studies (J. L. Xiong et al., 2018). In another study by Korgaonkar, Sarathchandra, Ponnusamy, and Karunakaran (2017), a toxin binder was also used in the diet of cows and buffaloes, and the transfer of AFM1 to milk was significantly reduced (Korgaonkar et al., 2017). The toxin adsorbent of this study contained compounds of phyllosilicates, mannan oligosaccharides and activated carbon (Korgaonkar et al., 2017). Oligosaccharides are extracted from the yeast wall of *Saccharomyces cerevisiae* (Mustafa et al., 2018; Yenice et al., 2015), contributing to increased immunity and improved digestive system functioning (Mustafa et al., 2018). In the study of Intanoo et al., three yeast species *Pichia kudriavzevii, Kluyveromyces marxianus,* and *K. marxianus* were investigated to reduce aflatoxin M. All three species were effective, but *Kluyveromyces marxianus*, and *K. marxianus* were most effective (Intanoo et al., 2020).

In the study conducted by Naveed et al. (2018), three binder toxin T5X, Mycosorb and Fixer Viva was used (Table 2). The most significant effect was observed regarding Mycosorb (Naveed et al., 2018). This composition contains the wall of Sacchromyces cerevisiae (Naveed et al., 2018). Studies indicate that this binder is more effective than inorganic binders (Naveed et al., 2018). T5X is a mixture of an organic and non-organic compound and Fixer Viva is a non-organic compound (Naveed et al., 2018). Conversely, contrasting results were noted in the study by Kutz et al. (2009). Three types of adsorbents were used in the ration of AFB1-contaminated animals for 4 periods of 7 days. Positive effects were observed with NovasilPlus, whereas no effect was observed with MTB-100 (Kutz et al., 2009). NovasilPlus and Solis are hydrated sodium calcium aluminosilicates, whereas MTB-100 contains modified yeast cell culture(Kutz et al., 2009). Similar outcomes were reported in another study. In the study conducted by Rojo et al. (2014) no positive effects of feeding with yeast walls were seen (Rojo et al., 2014). It is likely that, this type of commercial toxin binder, which comprises modified yeast cell culture, requires reevaluation in its structure and formulation. It is also worth mentioning that the effectiveness of AFM1 prevention agent depends on many factors, including its concentration, treatment duration, and AFB1dose. In Kutz et al.'s study (2009), the concentration of MTB-100 was 0.5%. In another study, two groups were given 10 g and 50 g per cow of MTB. Positive effects were observed in the dose of 50 g (Moran et al., 2013).

In a study, lactating cows were given 1725 μg of AFB1 for 30 days. One group was given AFB1 plus clay (sodium bentonite), while the other group received AFB1, clay, and fermentation products from *Saccharomyces cerevisiae*. The effectiveness of both methods in the study by Jiang et al. (2018), can be seen in Table 1(Jiang et al., 2018a). Similar to this experimental study, it was observed in Mirzaei's study. In a recent investigation, cows were deliberately fed with AFB1. In this 42-day study, the animals' feed was monitored for AFB1 levels weekly, ranging from 12 to 16 ppt (Mirzaei et al., 2020). The *S. cerevisiae* group exhibited the most favorable response in this study. The yeast's cell wall enhances rumen functional ecosystems, but its exact action mechanism needs more research (Mirzaei et al., 2020).

In the Firmin et al. (2011) study, food rations contaminated with AFB1 were used along with genetically modified yeast. It was observed that the amount of faecal excretion of both AFB1 and AFM1toxins has increased, this emphasizes the reduction of absorption (Firmin et al., 2011). Furthermore, in a 28-day study, animals were fed animal feed with AFB1 and several types of yeast. The yeasts used were K. marxianus CPY1 (K1Y), K. marxianus RSY5 (K2Y) or P. kudriavzevii. The greatest effect was observed in feeding with K. marxianus(Intanoo et al., 2020).

In a study conducted by Ali Tipu et al. (2021), the category of food additives was investigated. They found that including calcium propionate in the diet of livestock resulted in a reduction in the transfer of AFM1 to milk without altering the quantity or composition of milk (Tipu et al., 2021). The authors considered a 0.5% dose of calcium propionate to be safe (Tipu et al., 2021). Calcium propionate is one of the food preservatives (El Houssni, Khedid, Zahidi, & Hassikou, 2023). Furthermore, in the study by Galvano et al. (1996), the use of rations containing 2 % of activated carbon led to a decrease in the amount of toxins in milk. The affinity of activated carbon to aflatoxins was observed in this study both in vitro and in vivo (Galvano et al., 1996).

Three studies investigated the efficiencyof vaccination to reduce AFM1 (Giovati et al., 2014; Polonelli et al., 2011; Roshdy et al., 2020). Vaccination of livestock against AFB1leads to the reduction of AFM1. The antibodies produced during vaccination effectively captured AFB1, leading to a reduction in AFM1 level (Giovati et al., 2014). Toxicity evaluation was also done regarding the antibody produced against aflatoxin B1. Toxicity evaluation was also done regarding the antibody produced against aflatoxin B1. The produced antibody did not show toxicity in liver cells. Also, no mutagenic effects were observed in *Salmonella typhimurium* strains (Polonelli et al., 2011). In the study by Roshdy et al. (2020), a booster dose administered three weeks after the initial dose, and more effects were observed (Roshdy et al., 2020).

### 4.3 Interventions made on milk

**Fig. 3:**
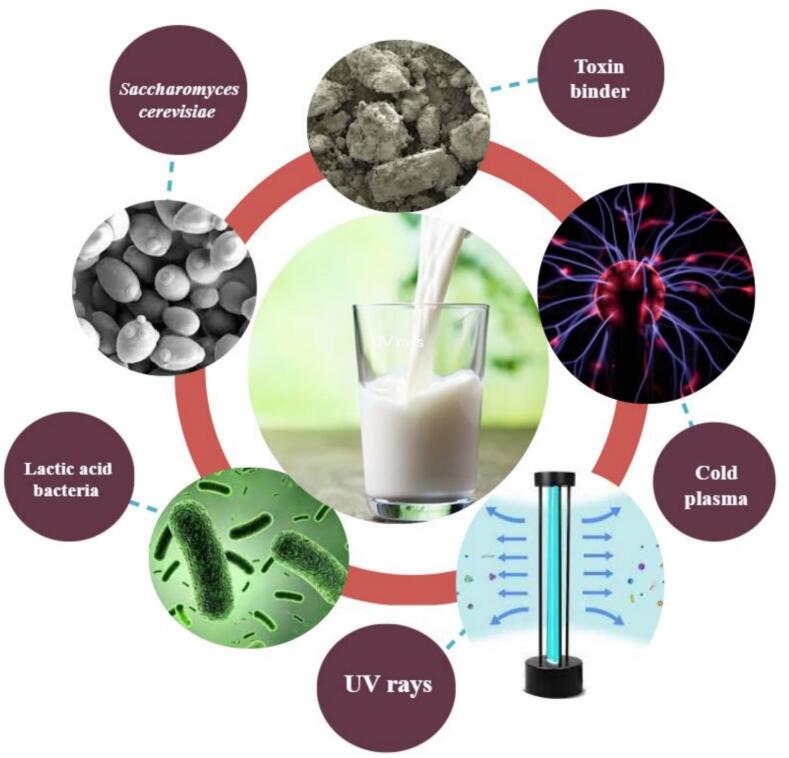
Common interventions performed on milk to reduce AFM1 in milk.

A series of interventions to reduce the AFM1 in milk have been conducted directly on the milk (Fig. 3). In this type of study, milk containing AFM1 is usually exposed to microorganisms such as bacteria and yeast at a temperature of 37 °C for varying durations. Subsequently, the samples are centrifuged, and the supernatant is assessed for the presence of AFM1, which is then compared with control groups (Corassin, Bovo, Rosim, & Oliveira, 2013).

In Ahmad's study, contaminated milk was incubated with *Lactobacillus acidophilus* bacteria. A significant amount of mycotoxin was reduced. Mycotoxin adhered to the bacterial wall at optimal temperature and pH (Ahmad, Samir, Mustafa, & Saadi, 2022). In the Bovo et al.(2012) study, various species of lactic acid bacteria were exposed to contaminated milk, and the most effective species was observed with *Bifidobacterium lactis*(Bovo, Corassin, Rosim, & de Oliveira, 2013). In the study of Kuharić et al. (2018), the bacteria species *L. plantarum* binds to AFM1 up to 95%, and it is possible to clean milk from AFM1 without the need for special skills (Kuharić et al., 2018). Similar to the results of this study, it was observed in the study of Khadivi, Razavilar, Anvar, and Akbari-Adergani (2020). *L. plantarum* bacteria led to 89% toxin reduction in this study(Khadivi et al., 2020). In Ismail et al. (2016) study in Brazil, milks were incubated with five models of three species of lactic acid bacteria and yeast. The groups were defined as follows: three groups of bacteria, respectively, *Lactobacillus plantarum*, *Lactobacillus helveticu*, *Lactobacillus lactic,* the fourth group is yeast and the fifth group is a mixture of bacteria and yeast. In the *Lactobacillus helveticu* group, the highest effect was observed (Ismail et al., 2017). In the Kabak et al.(2008) study, four species of lactic acid bacteria L. *acidophilus*, *L. acidophilus,* L. *acidophilus* and *L. rhamnosus* and two species of Bifidobacterium including *B. bifidum* and *B. bifidum* were incubated with milk containing AFM1(Kabak & Var, 2008). In this study that was conducted in 2008, this method is not declared to be an effective method (Kabak et al., 2008). The opposite result of this study was seen in Khadivi et al. (2020) study*. Lactobacillus rhamnosus* bacteria species with a high percentage led to the reduction of toxin in the incubated milk (Khadivi et al., 2020).

In Martínez, Magnoli, González Pereyra, and Cavaglieri (2019) study, six species of lactic acid bacteria and yeast were incubated with milk containing toxin and the power of toxin decomposition was investigated (Martínez et al., 2019). In this study, in addition to the cleaning power of these species, the toxicity of the resulting metabolites was investigated with the toxicity test of male *Artemia salina*. The resulting metabolites were non-toxic after 24 h of exposure to *P. acidilactici* and L. *rhamnosus*, but after 48 h the metabolites were toxic and led to the death of Artemia larvae (Martínez et al., 2019). Similarly, in another study, skimmed milk was treated with two species of bacteria, *Bifidobacterium lactis*, and *Lactobacillus bulgaricus*, along with AFM1. Both bacteria were capable of degrading AFM1 within 30 min at 4 °C (Namvar Rad, Razavilar, Anvar, & Akbari-Adergani, 2018).

In Corassin, Bovo, Rosim, Oliveira, and d. (2013) study, *Saccharomyces cerevisiae* alone and in combination with three species of lactic acid bacteria were exposed to milk contaminated with AFM1. The binding rate of lactic acid bacteria with AFM1 was 11%, the binding rate of yeast was 90%, and the binding rate of the mixture of bacteria and yeast was 91% (Corassin et al., 2013).

In addition to biological interventions, certain physical interventions were carried out. In the study of Hernández-Falcón et al. (2018), milk was subjected to ultrasound for durations of 10 and 15 min. Exposure for 10 min was effective in reducing the AFM1. In addition, no changes were observed in the milk's specification following 10 min of ultrasound treatment, whereas alterations were noted after 15 min (Hernández-Falcón et al., 2018). In the extracted data, only two studies dealt with the effect of cold plasma on the rate of AFM1decomposition in contaminated milk (Nguyen et al., 2022; Nikmaram et al., 2023). The evaluation of the toxicity of the AFM1 was also investigated with the *Artemia salina* test. It was observed that contaminated milk exposed to cold plasma, the survival rate of Artemia larvae increases up to 83.9% (Nguyen, Palmer, Phan, et al., 2022). Cold plasma is a new technology that works in a short period of time with low energy and without the need for a vacuum and pressure device. Can be done at room temperature (Nikmaram et al., 2023). The amount of AFM1 reduction was dependent on the duration of exposure.

In an earlier study by Yousef (1986), contaminated milk was exposed to UV rays, with the most significant effect observed at a temperature of 25 °C for 20 min (Yousef & Marth, 1986). In a study resembling Yousef's, the toxin level decreased by 50% in contaminated milk (initially at 1 μg/L) after exposure to UVC for 20 min (Nguyen et al., 2022). Typically, milk safety should be verified post-radiation. In another study, recombinant peroxidases were used for direct degradation of aflatoxin in milk. Milks contaminated with AFM1 and peroxidase were exposed to liver cells, compared to milks containing AFM1, the survival of cells were increased (Liu et al., 2023). However, further safety evaluations of the exposed product are necessary.

Some interventions on milk involved chemical treatments. In most studies, milk was incubated with lactic acid bacteria. It was incubated with absorbent only in one study. In this recent study, raw milk containing aflatoxin M was incubated with Kaolin and Ca-bentonite adsorbents in three concentrations of 5, 10, and 20 g. Both absorbents exhibited positive and dose-dependent effects (Moussa, Sobeih, Al-Hawary, Elkassas, & Barakat, 2020). The authors did not explain the mechanism of action, but stated that no nutritional changes were observed in the milk (Moussa et al., 2020). In another study, AFM1-contaminated milk was incubated with cyclodextrin, which is a common compound in the food industry, which led to some reduction (39.1%) of the AFM1(Šimko & Kolarič, 2022). Cyclodextrin is easily separated from milk via centrifugation (Šimko et al., 2022).

## 5 Conclusion

Interventions aimed at reducing AFM1 levels in milk can be categorized into three main groups. Some interventions focus on livestock fodder. In this regard, fodder is exposed to ammonia. These studies are old and have not been repeated in recent years. Another significant group of interventions involves the use of feed additives, including clay, algae, yeast, or a combination thereof, in animal feed. This approach has been extensively explored in numerous manuscripts and has shown promising results, with the combination of clay and yeast demonstrating particularly effective responses. In three manuscripts, vaccination of cows was used to control AFM1 in milk, which was reported to be moderately effective. A third category of interventions involves direct treatment of milk. This includes exposing milk to probiotic bacteria, clay, UV rays, and cold plasma. Exposure to UV rays and cold plasma should ensure the safety of milk after exposure. In this regard, more complete studies are required in the form of animal toxicity tests. Overall, among the interventions, the use of feed additives in animal diets appears to be the most effective, practical, and economical method.

## CRediT authorship contribution statement

**Yeganeh Mazaheri:** Writing – original draft. **Nabi Shariatifar:** Methodology. **Alireza Bakhtiyari:** Data curation. **Zahra Hadian:** Methodology. **Nader Akbari:** Writing – review & editing. **Narges Abdoli:** Writing – original draft. **Parisa Sadighara:** Writing – original draft.

## Declaration of competing interest

The authors declare that they have no known competing financial interests or personal relationships that could have appeared to influence the work reported in this paper.

## Data Availability

The data that has been used is confidential.
